# Immune-mediated cochleovestibular dysfunction: clinical spectrum from isolated inner-ear disorders to systemic autoimmune diseases and therapeutic strategies

**DOI:** 10.3389/fimmu.2026.1761486

**Published:** 2026-03-25

**Authors:** Yahui Wu, Jianlei Zhao, Junhu Tai, Xian Jiang, Xudong Li

**Affiliations:** 1 Department of Otolaryngology Head and Neck Surgery, Affiliated Hospital of Yanbian University, YanJi, China; 2 Department of Plastic Surgery, Affiliated Hospital of Yanbian University, YanJi, China; 3 Department of Otolaryngology Head and Neck Surgery, Xiamen Medical College, Xiamen, China

**Keywords:** autoantibodies, autoimmune diseases, immunopathology, vertigo, vestibular diseases

## Abstract

Immune-mediated cochleovestibular dysfunction has gained recognition as an important yet frequently overlooked entity in recent decades. These disorders—ranging from isolated inner-ear syndromes to cochleovestibular manifestations of systemic autoimmune diseases—exhibit humoral or cellular immune attacks on inner-ear structures, commonly accompanied by microvascular injury and inflammatory cascades. Despite increasing awareness, the precise pathophysiological mechanisms remain incompletely understood for most conditions, and diagnostic and therapeutic approaches vary considerably. This narrative review summarizes current evidence on immune-mediated cochleovestibular disorders, dividing them into two main categories (1): primary Isolated disorders (delayed endolymphatic hydrops, bilateral vestibulopathy, and Ménière’s disease with established or suspected autoimmune features) (2) cochleovestibular manifestations of rheumatologic diseases (systemic lupus erythematosus, multiple sclerosis, autoimmune thyroid disease, Behçet’s disease, Vogt-Koyanagi-Harada disease, psoriasis, Cogan’s syndrome, Susac syndrome, Sarcoidosis, Rheumatoid arthritis, Necrotizing vasculitides with polyangiitis and Giant cell arteritis). We examine their clinical features, proposed immune and microvascular mechanisms, diagnostic evaluation, and current management strategies, with particular emphasis on immunomodulatory and immunosuppressive therapies. Systemic corticosteroids at high doses are the primary treatment for most of these disorders, though the ideal duration, tapering protocols, and indications for steroid-sparing medications differ significantly across various syndromes. Evidence supporting many adjunctive therapies is limited or conflicting, underscoring the need for higher-quality clinical trials. Early recognition and prompt immunomodulatory treatment can often reverse or stabilize symptoms in immune-mediated cochleovestibular dysfunction. This review offers a clinically oriented synthesis of current evidence, elucidating the complex immunological underpinnings and the corresponding therapeutic landscape of these disorders. By integrating otologic and rheumatologic perspectives, we aim to heighten awareness, promote earlier diagnosis, and inform more effective treatment of patients presenting with vertigo, hearing loss, or imbalance suggestive of immune-mediated inner-ear pathology.

## 1 Introduction

Immune mechanisms in the pathogenesis of cochlear-vestibular diseases have gained increasing attention in recent years. Various vestibular pathological changes, whether occurring as a primary disorder or associated with a systemic autoimmune condition, can result from abnormal immune activity. While the precise cause and mechanisms remain uncertain, recent studies suggest that both humoral and cellular immunity, along with microvascular damage and inflammatory cascades, can target inner ear structures, resulting in functional impairments and clinical manifestations such as vertigo, balance disorders, and hearing loss. This article systematically reviews the current understanding of immune-related vestibular dysfunction, categorizing these diseases into two major types: isolated cochlear-vestibular disorders and syndromes associated with systemic autoimmune diseases. A comprehensive list of the two main types of vestibular dysfunction and their primary pathophysiological mechanisms is provided in **Table 1**. The goal is to provide a clinically oriented overview based on contemporary literature, addressing their diagnosis, pathophysiology, and management.

**Table 1:** Summary of vestibular dysfunction caused by different immune diseases.

Classfication	Disease	Mechanisms	Key diagnostic markers	References
Isolated Diseases	Delayed Endolymphatic Hydrops (DEH)	Viral infection-induced microvascular damage disrupts endolymphatic fluid absorption, causing hydrops.	Imaging: 3D-FLAIR MRI evidence of endolymphatic hydrops. Electrophysiology: Reduced VEMP amplitude on the affected side.	(1–17)
	Bilateral Vestibulopathy (BVP)	Immune-mediated damage to bilateral vestibular organs or nerves, leading to dysfunction.	Clinical: Bilateral vHIT VOR gain < 0.6; significantly reduced bithermal caloric responses (sum of max peak slow phase velocities < 6°/s); decreased rotary chair gain; marked instability on posturography (Condition 5/6).	(18–34)
	Ménière’s Disease	Autoimmune-mediated microvascular injury causes endolymphatic hydrops and vestibular dysfunction.	Serology: Positive anti-HSP70 (68-kDa) antibodies; elevated proinflammatory cytokines (IL-1β, TNF-α); Clinical: Significant response to initial corticosteroid therapy.	(35–54)
Systemic Diseases	Systemic Lupus Erythematosus (SLE)	Immune complex deposition in microvessels of the inner ear causes inflammation and ischemia.	Laboratory: Positive Anti-dsDNA and Anti-Sm antibodies; decreased complement C3/C4 levels; presence of lupus anticoagulants, indicating microcirculatory risk.	(56–66)
	Multiple Sclerosis (MS)	Demyelination of vestibular pathways impairs balance and spatial orientation.	Imaging: Positive Oligoclonal bands (OCB); MRI evidence of demyelinating plaques in the brainstem; Electrophysiology: Prolonged VEMP latencies reflecting central conduction blocks.	(67–79)
	Autoimmune Thyroid Disease (Hashimoto’s Thyroiditis)	Autoantibodies induce microvascular inflammation in the inner ear, disrupting lymph flow and function.	Serology: Elevated Anti-TPO and Anti-Tg antibody titers; Clinical: Abnormal TSH and FT4 levels; Imaging: Diffuse hypoechogenicity or “thyroid inferno” sign on ultrasonography.	(80–94)
	Behçet’s Disease	Vasculitis affecting the inner ear vasculature leads to ischemic vestibular dysfunction.	Vestibular/VNG: Abnormal bithermal caloric tests; saccadic dysfunction on video nystagmography (VNG); Electrophysiology: Prolonged VEMP latencies or abnormal waveforms indicating vestibulospinal pathway involvement.	(95–100)
	Vogt-Koyanagi-Harada (VKH) Disease	CD4+ T-cell-mediated attack on melanocytes, affecting vestibular and cochlear functions.	Imaging: Elevated aqueous humor IL-6; CSF lymphocytosis; multifocal leakage on FA/ICGA; Clinical: High incidence (70%) of abnormal balance test results.	(101–111)
	Psoriasis	Dysregulated immune response disrupts inner ear immune homeostasis, contributing to vestibular dysfunction.	Quantitative: Significantly elevated DHI scores (7.70 ± 17.44); Vestibular: Reduced cVEMP amplitudes; vHIT: VOR gain deficits and frequent abnormal covert saccades (CS/CSD).	(112–124)
	Cogan’s Syndrome	Immune-mediated vasculitis affecting inner ear vessels, causing hydrops and vestibular dysfunction.	Clinical: Non-syphilitic interstitial keratitis (IK); acute Ménière-like vestibular crises; MRI evidence of membranous labyrinth hydrops.	(125–133)
	Susac Syndrome	Microvascular injury affecting the brain, retina, and cochleovestibular system, leading to vestibular symptoms.	Imaging: Pathognomonic “snowball” lesions on MRI; retinal branch artery occlusion (BRAO). Electrophysiology: Selective cVEMP dysfunction reflecting saccular ischemic injury.	(134–140)
	Sarcoidosis	Th17.1-driven inflammation induces “perineural cuffing” and axonal degeneration. Microvasculitis leads to secondary ischemic damage to vestibular organs.	Imaging: MRI shows leptomeningeal enhancement or nerve hypertrophy. Laboratory: CSF lymphocytic pleocytosis and elevated protein. Clinical: Vertigo, imbalance, or hearing loss	(141–159)
	Rheumatoid Arthritis(RA)	Type II collagen autoantibodies target the inner ear matrix, triggering inflammatory necrosis and mechanical canal collapse. Pro-inflammatory cytokines and microvasculitis induce oxidative stress and ischemic degeneration of vestibular receptors.	Serology: High titers of RF and ACPA; elevated acute-phase reactants (CRP and ESR). Clinical: Vestibular vertigo and joint involvement according to 2010 ACR/EULAR criteria.	(160–175)
	Granulomatosis with Polyangiitis(GPA)	The PR3-ANCA-mediated C5a-neutrophil amplification loop triggers necrotizing vasculitis within the vestibular microcirculation, leading to the selective destruction of type I vestibular hair cells. Furthermore, inflammatory occlusion of the labyrinthine artery induces ischemic necrosis and localized microhemorrhages within the vestibular system.	Serology: High prevalence of PR3-ANCA positivity (80%–90%). Clinical: Refractory otorhinolaryngological manifestations (e.g., chronic rhinosinusitis, serous otitis media) and disabling vertigo.	(176–183)
	Giant Cell Arteritis(GCA)	Stratified arterial wall inflammation triggers terminal vessel occlusion. Adventitial and medial injury disrupts structural integrity, while intimal hyperplasia drives luminal stenosis, leading to ischemic vestibulopathy and secondary otoconia displacement.	Imaging: Ultrasound “halo sign” or increased metabolic activity on PET/CT. Clinical: New-onset headache and jaw claudication.	(191–210)

3D-FLAIR MRI, 3-dimensional fluid-attenuated inversion recovery magnetic resonance imaging; ACPA, anti-citrullinated protein antibodies; ACR/EULAR, American College of Rheumatology/European Alliance of Associations for Rheumatology; ANCA, antineutrophil cytoplasmic antibodies; Anti-dsDNA, anti-double-stranded DNA; BPPV, benign paroxysmal positional vertigo; BVP, bilateral vestibulopathy; CRP, C-reactive protein; CSF, cerebrospinal fluid; cVEMP, cervical vestibular evoked myogenic potentials; DEH, delayed endolymphatic hydrops; ESR, erythrocyte sediment rate; GCA, giant cell arteritis; GPA, granulomatosis with polyangiitis; IL-1β/IL-6, interleukin-1 beta/interleukin-6; MS, multiple sclerosis; PR3-ANCA, proteinase 3-antineutrophil cytoplasmic antibody; RA, rheumatoid arthritis; RF, rheumatoid factor; SLE, systemic lupus erythematosus; TNF-α, tumor necrosis factor-alpha; vHIT, video head impulse test; VNG, video nystagmography; VOR, vestibulo-ocular reflex.

## 2 isolated cochlear-vestibular disorders

Isolated immune-mediated vestibular dysfunction encompasses delayed endolymphatic hydrops (DEH), bilateral vestibulopathy (BVP), and Ménière’s disease (MD). These disorders involve immune-mediated injury to the inner ear, resulting in vestibular symptoms, including vertigo and balance disorders. DEH typically follows severe hearing loss, BVP presents as bilateral vestibular dysfunction, and endolymphatic hydrops is the main underlying cause of Ménière’s disease, with immune mechanisms playing a critical role in its pathogenesis.

### 2.1 Delayed endolymphatic hydrops

Delayed Endolymphatic Hydrops (DEH) is an uncommon vestibular condition that develops after a latency period following significant sensorineural hearing loss, marked by the onset of membranous labyrinth hydrops (1–3). DEH is categorized into three types according to clinical manifestations: ipsilateral type (IDEH), in which vertigo occurs in the previously deaf ear; contralateral type with vertigo (CDEHwV), characterized by fluctuating hearing loss and vertigo in the unaffected ear, while the contralateral type without vertigo (CDEHwoV) involves only intermittent hearing loss in the opposite ear. A nationwide study in Japan found that among 662 DEH patients, 55.4% had IDEH, and 44.6% had CDEH, with a CDEHwV to CDEHwoV ratio of 70.8% to 29.2%. The disease is most common in individuals around 50 years old, with a slightly higher incidence in females (55.6%). The latency period for contralateral type (CDEH) (approximately 25–29 years) is significantly longer than for IDEH (approximately 22 years) (4).

The exact cause of DEH is still unknown, although it is believed to be closely linked to labyrinthine damage caused by viral infections. Schuknecht (5) and colleagues hypothesized that viral infections cause not only initial deafness but also damage the endolymphatic sac’s absorption function, ultimately triggering endolymphatic hydrops. Pathological studies indicate that in CDEH, the previously deaf ear shows pathological changes similar to viral labyrinthitis (e.g., measles and mumps), while the opposite ear exhibits features resembling Meniere’s disease. This implies that autoimmune mechanisms could be involved in its pathogenesis, potentially resembling “sympathetic ophthalmia.” (6, 7) Recent high-throughput RNA sequencing studies have further revealed that DEH is linked to neuropathy and autoimmunity, with cell adhesion processes contributing to disease development (8). Imaging studies using 3T-MRI gadolinium-enhanced scans have confirmed vestibular hydrops in the previously deaf ear of IDEH patients and cochlear hydrops in the opposite ear of CDEH patients, providing objective morphological evidence for diagnosing DEH (9, 10).

Management of DEH should be individualized according to clinical subtype and therapeutic response. Initial therapy prioritizes pharmacological interventions, such as diuretics, corticosteroids, and vasodilators (11). Patients refractory to oral medications may require second-line interventions, including intratympanic injections (gentamicin or dexamethasone) or middle ear pressure therapy (12, 13). Surgery is reserved for refractory cases that are non-responsive to conservative management. For refractory IDEH, vestibular ablation—performed via labyrinthectomy or vestibular neurectomy—or semicircular canal occlusion offers definitive symptom relief (14–16). Conversely, endolymphatic sac surgery is preferred for CDEH to preserve residual hearing in the only or better-hearing ear (11). Long-term data indicate a favorable prognosis, with approximately 70% of patients achieving sustained symptomatic relief. Hearing loss in the affected ear typically remains moderate; progression to profound deafness is rare (17).

### 2.2 Bilateral vestibulopathy

Bilateral vestibulopathy (BVP) is a chronic and progressive clinical disorder characterized by the profound impairment or loss of function in the peripheral labyrinths or the vestibular portions of the eighth cranial nerves bilaterally (18). It affects approximately 1.8 million adults worldwide and significantly elevates fall risk (19). In 20%–50% of idiopathic cases (idiopathic BVP, IBV), immune-mediated damage is identified as a primary etiological factor (20–22). The pathophysiology involves an intricate interplay between humoral immunity, microvascular compromise, and inflammatory cascades. Humoral responses induce the deposition of organ-specific IgG antibodies targeting antigens such as heat shock protein-70, type II collagen, and myelin protein P0. These antibodies synergize with T-cell-mediated immunity to attack vestibular end-organs (23–25). Additionally, microvascular damage—arising from localized vasculitis or labyrinthine artery occlusion—disrupts the blood-labyrinth barrier. This ischemia triggers the selective apoptosis of vestibular hair cells (26).

Precise diagnosis of immune-mediated BVP necessitates transitioning from conventional diagnostic metrics, such as a video head impulse test (vHIT) gain < 0.6 or caloric responses < 6°/s (27), to identifying the etiology through multidimensional molecular profiling. While definitive pathogenic mutations for immune-mediated BVP remain unidentified—unlike the Replication Factor C Subunit 1 (RFC1)-mediated Cerebellar Ataxia, Neuropathy, Vestibular Areflexia Syndrome (CANVAS)—genetic susceptibility likely predisposes individuals to immune triggering (28). Inner-ear-specific antibodies detected via Western blot represent pivotal diagnostic biomarkers. Furthermore, BVP frequently serves as a localized manifestation of systemic immune dysfunction, with 23.4% of patients exhibiting comorbid systemic autoimmune disorders. Consequently, screening for ESR, ANA, and antiphospholipid antibodies offers critical differential diagnostic value. These markers help identify systemic conditions such as Cogan’s syndrome, systemic lupus erythematosus (SLE), or polyarteritis nodosa (29).

Management of immune-mediated BVP requires a paradigm shift from functional compensation to homeostatic restoration. Based on immunopathological evidence, systemic corticosteroids (e.g., methylprednisolone) or immunosuppressants should be established as primary first-line therapies (30). Early intervention prevents the transition from reversible inflammation to irreversible fibrosis. Furthermore, vestibular recovery positively correlates with autoantibody seroconversion. In contrast, central compensation-based vestibular rehabilitation is strictly a second-line adjuvant measure (31). While noisy galvanic vestibular stimulation (nGVS) and vestibular implantation may improve dynamic balance, they are pathophysiologically non-specific substitution strategies (32–34). These methods do not halt primary immune destruction and should be reserved as salvage measures for refractory cases during the stable phase of immune activity.

### 2.3 Ménière’s disease

Menière’s disease (MD) is an idiopathic, recurrent cochleovestibular disorder defined by the clinical tetrad of rotational vertigo, fluctuating sensorineural hearing loss, ipsilateral tinnitus, and aural fullness (35). Epidemiology reveals a peak onset between 40 and 60 years of age, a slight female predominance, and a familial predisposition in approximately 10% of cases. Despite global variations in prevalence due to diagnostic criteria (e.g., 13.1 vs. ~190 per 100,000 in the UK and USA, respectively), the severe functional impairment imposed by MD remains undisputed (36–38). Pathophysiologically, the hallmark of MD is endolymphatic hydrops—an abnormal accumulation of endolymph within the membranous labyrinth (39). This pathological pressure elevation mechanically compromises vestibular hair cells and neural transmission, causing vestibulo-ocular reflex (VOR) deficits and spatial disorientation, while simultaneously establishing the physical substrate for progressive tissue degeneration (40).

The disruption of immune homeostasis within the endolymphatic sac (ES) microenvironment underpins the pathology of endolymphatic hydrops (EH). This process is driven by the skewed differentiation of CD4+ T cell subsets (41). Notably, aberrant humoral immunity mediated by Th2 cells plays a central role in this regulatory network. Bidirectional Mendelian randomization studies have established a causal link between allergic conditions (asthma, allergic rhinitis, eczema) and MD, highlighting a genetic susceptibility linked to atopy (42). Molecularly, Th2 polarization upregulates cytokines like IL-4, promoting IgE synthesis by B cells and CD23 receptor overexpression on inner ear hair cells. This facilitates transepithelial IgE transport and mast cell degranulation, leading to altered vascular permeability and EH (43). Concurrently, Th1 cells drive a sterile inflammatory response that constitutes a secondary insult. Proteomic analyses of endolymphatic sac luminal fluid (ELF) from MD patients reveal elevated levels of TNF-α, IFN-γ, and IL-6, confirming a localized inflammatory state. Specifically, IFN-γ accumulation inhibits epithelial sodium channel (ENaC) activity, disrupting electrolyte homeostasis (44). Furthermore, the inflammatory mediator IL-1β upregulates glutaminase (GLS), inducing excessive glutamate release. This triggers an “inflammation-excitotoxicity” cycle, impairing vestibular neuron function (45). Additionally, immune dysregulation involves a skewed Th17/Treg ratio and autoinflammatory mechanisms. Reduced Treg cell counts in MD patients suggest compromised immune tolerance. Moreover, elevated serum G-CSF, IL-8, and HGF levels correlate with neutrophil extracellular trap (NET) formation. NET release causes tissue damage and exposes autoantigens, potentially triggering secondary autoimmunity and inner ear fibrosis that exacerbate endolymphatic malabsorption (46).

Clinical management of MD is transitioning from empirical protocols toward molecularly driven precision interventions. Systemic pharmacotherapy remains the mainstay of initial treatment, encompassing betahistine for microcirculatory enhancement, diuretics for osmotic control, and context-specific antiviral applications. Beyond broad-spectrum anti-inflammation, corticosteroids—utilized as first-line systemic and local agents—exert their therapeutic effects by pleiotropically restoring inner ear immune homeostasis. This restoration involves the genomic inhibition of the NF-κB pathway, which effectively halts pro-inflammatory cytokine cascades at the transcriptional level (47–49). Simultaneously, these agents fortify the blood-labyrinth barrier by upregulating tight junction proteins, such as Occludin and Claudin-5, thereby obstructing systemic insults across both physical and immunological dimensions. Furthermore, corticosteroid-mediated activation of mineralocorticoid receptors modulates ion transporters—including AQPs and ENaC—to directly resolve endolymphatic immuno-osmotic imbalances (50).

For refractory cases resistant to conventional therapy, precision medicine is identifying targeted molecular breakthroughs. TNF-α inhibitors mitigate inflammatory damage through dual transcriptional inhibition and protein neutralization, thereby facilitating barrier repair, hydrops resolution, and the re-establishment of immune stability (51, 52). Concurrently, emerging agents such as SPI-1005, an NLRP3 inflammasome inhibitor, and OTO-104, a sustained-release hydrogel formulation, exhibit optimized pharmacokinetic and immunomodulatory profiles (53, 54). While evidence from randomized controlled trials remains nascent, integrating molecular phenotyping with core outcome sets promises to facilitate the clinical implementation of personalized, precision immunomodulatory strategies for MD.

## 3 Systemic immune disorders and vertigo

**Figure 1:**
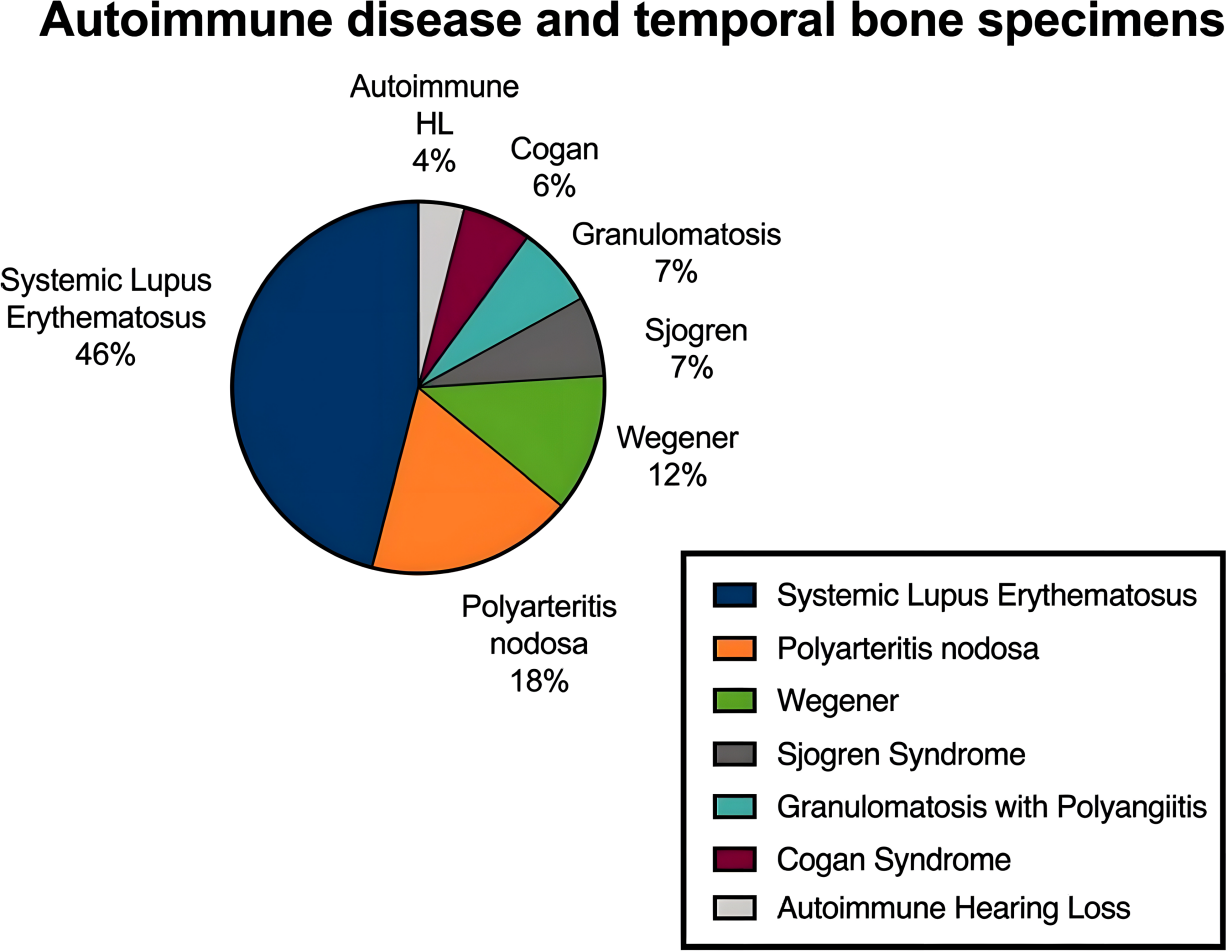
Autoimmune disease and temporal bone specimens. Reproduced from Di Stadio A, Ralli M. Systemic Lupus Erythematosus and hearing disorders: Literature review and meta-analysis of clinical and temporal bone findings. *Journal of International Medical Research.* 2017;45(5):1470-1480. doi:10.1177/0300060516688600, with permission from SAGE Publications.

Vestibular symptoms of systemic immune origin frequently occur in the context of autoimmune disorders such as systemic lupus erythematosus (SLE), multiple sclerosis (MS), Hashimoto’s thyroiditis, Behçet’s disease, Sarcoidosis, Rheumatoid arthritis(RA), Necrotizing vasculitides with polyangiitis(GPA) and Giant cell arteritis(GCA). These diseases lead to vestibular dysfunction through immune-mediated microvascular damage or immune cell attacks on the inner ear. Systemic diseases often involve widespread immune system damage, affecting multiple organs, with vestibular symptoms as one of the common clinical manifestations. Post-mortem temporal bone studies in individuals with systemic autoimmune disorders have consistently demonstrated inflammatory lesions within the labyrinth (55) (**Figure 1**).

### 3.1 Systemic lupus erythematosus

Systemic Lupus Erythematosus (SLE) is a chronic autoimmune disorder characterized by severe immune dysregulation and the production of pathogenic autoantibodies, such as anti-dsDNA (56). Epidemiology demonstrates a predilection for women of Asian and African descent, with peak onset between 20 and 39 years of age. Approximately 28% of SLE patients manifest vertigo and labyrinthine dysfunction (57–59). These symptoms arise from immunopathological mechanisms targeting multiple levels of the vestibular and auditory systems. Mechanistically, humoral and cellular immune responses targeting vestibular end-organ antigens cause direct tissue injury (60). Concurrently, the deposition of circulating immune complexes in inner ear microvessels triggers vasculitis, compromising labyrinthine perfusion (61). This immune dysregulation is corroborated by correlations between abnormal immune phenotypes and vestibular deficits observed on electronystagmography (EOG) (59). Additionally, antiphospholipid antibody (aPL)-mediated microvascular thrombosis drives Meniere’s-like manifestations (62). Post-mortem histopathology reveals selective degeneration of type I hair cells in SLE vestibular organs, accompanied by fibrosis and edema (55). In the auditory domain, SLE frequently induces sensorineural hearing loss (SNHL), primarily through immune complex-mediated vasculitis and thrombosis, damaging cochlear hair cells and the stria vascularis (63).

To address this immune-mediated damage, clinical management is shifting from broad-spectrum corticosteroids and immunosuppressants toward precision biologics. These agents target pathogenic checkpoints: Rituximab depletes B-cell clones to curb autoantibody production, while Anifrolumab antagonizes type I interferon receptors to block downstream inflammatory cascades. Beyond systemic remission, biologics preserve peripheral audiovestibular structure by ameliorating the inner ear microenvironment and reducing cytokine load (64). For refractory symptoms, transcutaneous vagus nerve stimulation (tVNS)—an emerging bioelectronic intervention—modulates the “neuro-immune axis.” Mechanistically, tVNS activates the hypothalamic-pituitary-adrenal (HPA) axis to release endogenous glucocorticoids and drives the cholinergic anti-inflammatory pathway to inhibit proinflammatory cytokines (e.g., TNF-α) via α7nAChR receptors (65, 66). This endogenous suppression of the “cytokine storm” aligns with the molecular targets of biologics like Belimumab, providing a physiological rationale for personalized, sequential therapy in SLE-related ototoxicity.

### 3.2 Multiple sclerosis

Multiple sclerosis (MS) is a chronic immune-mediated disease of the central nervous system characterized by inflammatory demyelination and neurodegeneration. Its worldwide prevalence is estimated at 35.9 per 100,000 population, with well-documented latitudinal gradients (67–70). MS notably affects the vestibular system, with approximately 20%-35% of patients experiencing vertigo, 1%-17% experiencing hearing loss, and about 25% exhibiting sudden or progressive hearing decline (71–73).

Vestibular function tests provide objective electrophysiological evidence of central and peripheral involvement in MS. These deficits stem primarily from inflammatory demyelinating plaques and axonal degeneration driven by peripheral immune cell infiltration across the blood-brain barrier. Electrophysiologically, prolonged cVEMP latencies (p13 and n23)map directly to focal demyelinating lesions within the brainstem vestibular nuclei and the root entry zone of the eighth cranial nerve (74). At this junction, pathogenic Th1, Th17, and CD8+ T cells attack myelin autoantigens, causing demyelination that shifts neural transmission from efficient saltatory conduction to slow, dissipative continuous conduction. This microstructural failure manifests macroscopically as neural conduction delays (75). Peripheral vestibular involvement, such as unilateral semicircular canal dysfunction detected by electronystagmography (ENG/VNG), indicates that systemic proinflammatory cytokines (e.g., TNF-α, IL-6) and chemokines have infiltrated the inner ear. Entry via a permeabilized Blood-Brain Barrier or perivascular spaces induces local inflammation and microcirculatory disturbances (76, 77). Concurrently, systemic inflammation activates NLRP3 inflammasomes within resident microglia or macrophages. This triggers Caspase-1-dependent release of IL-1β and IL-18 cascades. Activated microglia release matrix metalloproteinases, further compromising vascular integrity. Meanwhile, NLRP3-mediated mitochondrial damage and oxidative stress (ROS/RNS) drive secondary degeneration and atrophy of vestibular end-organs (78). Additionally, posturography testing reveals central sensory integration deficits, reflecting immune-mediated damage to cerebellar peduncles and brainstem circuits. Beyond sustained glial activation, chronic CNS inflammation redistributes axonal ion channels (e.g., ASIC1, TRPM4, sodium channels). This ionic dyshomeostasis, combined with lost neuronal connectivity, disrupts the spatiotemporal integration of multisensory information, underpinning the progressive balance deterioration seen in MS (75).

Systematic vestibular rehabilitation promotes central compensation in multiple sclerosis. A recent meta-analysis pooling six randomized controlled trials (n=321) demonstrated clinically meaningful gains in postural control, dizziness severity, and fatigue levels compared with usual care or no intervention (79).

### 3.3 Autoimmune thyroid diseases

Autoimmune thyroid diseases (AITDs) arise from breakdown of immunological tolerance to key thyroid antigens, principally thyroid peroxidase (TPO), thyroglobulin (Tg), and the TSH receptor (TRAb). This leads to both humoral and cellular immune responses, which are manifested by elevated levels of corresponding autoantibodies (TgAbs, TPOAbs, TRAbs) in circulation and thyroid lymphocytic infiltration (80, 81). Among AITDs, Hashimoto thyroiditis represents the predominant form of autoimmune thyroid disease, affecting 5%–10% of the general population worldwide. It is more common in women, and its prevalence increases with age (82).

Among individuals with autoimmune inner ear disease, Hashimoto thyroiditis accounts for approximately 17.9% of cases presenting with predominant vestibular involvement (83). The mechanism of vestibular involvement is primarily immune-mediated, rather than solely driven by abnormal thyroid hormone levels. The following pathophysiological hypotheses have been proposed (1): Immune complexes deposit in the inner ear, altering endolymph composition and mimicking mechanical stimuli, which induce vertigo (84). (2) Autoantibodies induce microvascular inflammation in the endolymphatic sac, disrupting endolymph flow and leading to vestibular symptoms (85, 86).

Clinical observations further emphasize the central role of immune mechanisms. A recent pooled analysis of multiple studies by Lima et al. revealed a robust association between Hashimoto thyroiditis and benign paroxysmal positional vertigo (BPPV) (87). Miskiewicz Orczyk et al. also reported no direct correlation between vestibular function test results and thyroid hormone levels (88). Additionally, approximately 26.7% of Hashimoto’s patients exhibit both cochlear and vestibular dysfunction (89). Hearing loss is associated with positive antithyroid antibodies, but not with thyroid dysfunction (90). Notably, about 79% of patients with autoimmune thyroiditis and comorbid BPPV have normal thyroid function, suggesting that vestibular symptoms are more likely due to immune-mediated inflammatory responses that directly damage vestibular receptor cells (90, 91).

A systematic review proposed a three-phase intervention strategy for immune-mediated vestibular disorders: Phase 1: trial therapy with high-dose oral prednisone (50 mg/day for 1 week), with response criteria being a ≥15% improvement in hearing threshold or significant reduction in vestibular symptoms. Phase 2: A 4-week course of prednisone at 5–10 mg/day co-administered with levothyroxine (92, 93). Phase 3: vestibular-specific non-invasive brain stimulation for patients with residual symptoms. Reports also indicate that patients who underwent thyroidectomy experienced significant relief from vertigo symptoms, suggesting that surgery provides a practical therapeutic pathway for otherwise untreatable severe vestibulopathy (94).

### 3.4 Behçet’s disease

Behçet’s disease (BD) is a chronic, recurrent inflammatory vasculitis that involves multiple systems, primarily affecting the skin, mucous membranes, eyes, joints, nervous system, and blood vessels. BD is more widespread and severe along the “Silk Road” region (Mediterranean, Middle East to Far East), with a global prevalence of approximately 10.3 per 100,000. It mainly affects young and middle-aged individuals, the mean age at first symptom ranges from 22 to 31 years, with diagnostic confirmation typically occurring between 31 and 33 years (95). Vestibular dysfunction is a major neurological manifestation of BD. Choung et al. observed significant vestibular dysfunction in patients through the bithermal caloric test (96). Kulahli et al. also observed abnormal saccades using video nystagmography, suggesting involvement of the central vestibular pathways (97). Recent studies using vestibular evoked myogenic potentials (VEMP) have shown prolonged latencies or abnormal waveforms in BD patients, suggesting that inflammation may affect the vestibulospinal pathways and brainstem-related structures (98, 99). Two main hypotheses currently explain the mechanism of vestibular damage in BD: the first is the vasculitic hypothesis, which suggests that BD involves the labyrinthine artery, cochlear artery, or anterior vestibular artery, causing ischemic damage to the cochlea and vestibular end organs (96). The second is the central dysfunction hypothesis. Erbek et al. found prolonged cVEMP latencies, further supporting impaired central integration in the brainstem, particularly in the vestibulocollic reflex pathway (99). This suggests that vasculitis and demyelination in BD may directly affect the vestibular nuclei and their central connections.

The treatment strategy for Behçet’s disease has shifted from traditional immunosuppressive therapy to targeted therapies targeting specific inflammatory pathways. In addition to corticosteroids, azathioprine, cyclosporine, and other immunosuppressive agents, biologic drugs have shown significant efficacy in treating refractory cases. Recent years have seen significant progress in targeted therapies aimed at the interleukin-1 (IL-1) pathway. Studies have shown that the IL-1 receptor antagonist anakinra effectively controls refractory Behçet’s disease symptoms. Additionally, anti-IL-1 therapy has demonstrated good safety and efficacy in multicenter studies (100).

### 3.5 Vogt-Koyanagi-Harada

Vogt–Koyanagi–Harada (VKH) disease is a systemic autoimmune disorder primarily directed against melanocyte-containing tissues, including the uvea, inner ear, skin, and meninges. Its primary mechanism involves an autoimmune response targeting melanocytes that express tyrosinase family proteins (101). The disease shows substantially higher prevalence among pigmented populations, particularly individuals of Hispanic, Native American, and Asian ancestry, with a higher incidence in women (102). Vestibular and auditory dysfunction are common early signs of VKH, including vertigo, tinnitus, and sensorineural hearing loss. Studies show that, among 20 VKH patients, approximately 25% exhibit vestibular symptoms (e.g., dizziness or vertigo), and 70% show abnormal balance test results, indicating peripheral vestibular dysfunction (103).

Immunopathological studies have established that the core pathogenesis of VKH involves CD4^+^ cytotoxic T lymphocytes targeting and attacking melanocytes (104, 105). Within the eye, the autoimmune process is marked by infiltration of activated CD4^+^ and CD8^+^ T cells into the aqueous humor, together with increased concentrations of proinflammatory cytokines such as IL-6, both of which correlate closely with clinical disease severity (106). Tyrosinase has been identified as a key autoantigen that specifically activates lymphocytes from VKH patients and induces VKH-like disease in animal models (107, 108). Additional studies highlight the central roles of IFN-γ and IL-6 in driving the inflammatory cascade, while metabolomic analyses reveal dysregulated amino acid and fatty acid metabolism in patients’ body fluids, implicating metabolic abnormalities in VKH onset and progression (109, 110).

VKH is primarily managed with high-dose systemic corticosteroids to achieve rapid remission, followed by a slow taper maintained for a minimum of 6 months. Short-term regimens (<6 months) carry a recurrence rate of up to 58.8%, whereas prolonged standardized therapy (≥6 months) markedly lowers the relapse rate to 11.1% (111). For patients who are corticosteroid-intolerant or require long-term maintenance, additional immunosuppressive agents (e.g., azathioprine or mycophenolate mofetil) are recommended.

### 3.6 Psoriasis

Psoriasis is a chronic immune-mediated systemic inflammatory disorder affecting roughly 2% of the global population. It typically manifests as sharply demarcated erythematous plaques covered with silvery-white scales, predominantly on the skin and scalp, but it is now established as a systemic disease affecting multiple organ systems (112–114). Psoriasis pathogenesis involves complex interactions among genetic, environmental, and immunological factors, with the exact mechanisms still incompletely elucidated. T-cell-mediated autoimmunity and dysregulated keratinocyte proliferation represent the core pathologic processes (115).

Dizziness and vestibular dysfunction occur frequently among individuals with psoriasis. Patients with psoriasis had a mean Dizziness Handicap Inventory (DHI) score of 7.70 ± 17.44—significantly higher than healthy controls, exceeding the minimal clinically important difference (MCID), and marking the first systematic documentation of DHI abnormalities in this population (116). Additional vestibular testing showed reduced p13-n23 amplitudes on cervical vestibular evoked myogenic potentials (cVEMP), indicating potential impairment of the vestibulocollic reflex pathway (117). Video head impulse testing (vHIT) revealed no significant difference in mean vestibulo-ocular reflex (VOR) gain across semicircular canals between groups; however, abnormal VOR gain in the right anterior canal was significantly more frequent in psoriasis patients (P = 0.047). Saccade analysis further identified significantly higher rates of abnormal covert saccade sequences (CS) in the left posterior canal plane (P = 0.037) and abnormal covert saccade direction (CSD) in the right anterior–left posterior (RALP) plane (P = 0.035) (116).

Vestibular dysfunction in psoriasis is thought to arise from disrupted immune homeostasis within the inner ear. The endolymphatic sac—a key inner-ear structure with antigen-presenting and local antibody-producing capabilities—can become dysfunctional in systemic autoimmune states. Harris proposed that the blood–labyrinth barrier, analogous to the blood–brain barrier, maintains inner-ear immunoglobulin homeostasis and allows the inner ear to react to systemic immune dysregulation (118). In systemic autoimmune diseases like psoriasis, abnormal antibody responses in the endolymphatic sac may disturb inner-ear immune homeostasis, resulting in vestibular dysfunction and potentially explaining clinically observed caloric test abnormalities (119).

Psoriasis offers a wide range of therapeutic options, with treatment selection guided primarily by disease severity and patient-specific factors. Topical therapies remain the preferred initial approach, particularly for mild psoriasis, where agents such as Roflumilast cream or nanoparticle-based biologics are commonly applied (120, 121). For moderate-to-severe psoriasis, phototherapy (e.g., narrowband UVB) and biologics (e.g., etanercept and adalimumab) are widely used and can markedly improve symptoms and reduce inflammation (122). Biologic agents are particularly valuable in patients whose disease remains refractory to conventional systemic treatments. Small-molecule drugs, such as phosphodiesterase-4 (PDE4) inhibitors, represent another effective option, providing favorable safety profiles and convenient administration. In addition, research on stem cell therapy and microbiome modulation is progressing, and although both remain experimental, they may offer promising therapeutic avenues in the future (123, 124).

### 3.7 Cogan’s syndrome

Cogan’s syndrome is a rare systemic vasculitis first defined by David G. Cogan in 1945 by the combination of non-syphilitic interstitial keratitis and vestibulo-auditory symptoms (125, 126). The underlying mechanism remains unclear, but the disease is widely regarded as an immune-mediated vasculitis targeting vessels of varying caliber, with autoantibodies targeting corneal, inner-ear, and endothelial antigens (127). Its incidence is unknown, with fewer than 250 documented cases in the global literature to date. It primarily affects young adults (20–30 years), is rare in children, and exhibits no gender bias or familial aggregation. Diagnostic delay averages 12–24 months, with ocular (41%) or vestibulo-auditory (43%) symptoms most frequently serving as the initial manifestation (128, 129). Patients typically present with acute Ménière-like vestibular crises (vertigo, tinnitus, and aural fullness) accompanied by nystagmus and ataxia. Although acute labyrinthine symptoms often resolve within days, progressive sensorineural hearing loss—unilateral or bilateral—commonly ensues (130). Imaging and histopathology provide additional mechanistic insights: MRI may show membranous labyrinth hydrops, whereas acute-phase specimens demonstrate brownish pigment deposition and inflammatory infiltrates in vascular-rich areas (e.g., cochlear lateral wall and spiral ganglion) (131, 132). Such lesions can impair endolymphatic circulation and resorption, thereby causing endolymphatic hydrops and the resultant auditory-vestibular symptoms.

Systemic corticosteroids are the first-line treatment, initiated orally at 1 mg/kg/day (maximum 60 mg/day) and, when severe, preceded by intravenous methylprednisolone pulse therapy (15 mg/kg/day for 3 days, maximum 1 g per dose). The optimal duration of corticosteroid therapy in Cogan’s syndrome remains undefined; current regimens are therefore adapted from protocols established for systemic vasculitides. Maintenance prednisone doses >5–7.5 mg/day should be avoided except during planned tapering. Immunosuppressive or biologic agents are indicated for steroid-dependent disease, frequent relapses, or intolerable corticosteroid-related adverse effects. Conventional immunosuppressants (methotrexate, azathioprine, mycophenolate mofetil, cyclophosphamide) and biologic agents—infliximab being preferred in cochleovestibular involvement—are both effective options (133).

### 3.8 Susac syndrome

Susac syndrome, originally described by John Susac in 1979, is an uncommon autoimmune microvasculopathy characterized by the classic triad of brain involvement, cochlear hearing loss, and retinal branch artery occlusions (134, 135). Fewer than 500 cases have been reported worldwide, with a marked female predominance (female-to-male ratio ≈ 3:1) in patients aged 20–40 years (136). The histopathological hallmark is arteriolar endothelial injury with basal lamina disruption. This autoimmune microangiopathy simultaneously targets the brain, cochleovestibular system, and retinal arterioles, producing the syndrome’s characteristic clinical phenotype (137).

In Susac syndrome, vestibular symptoms are characterized by persistent postural instability rather than classic rotational vertigo. Vestibular testing confirms predominant saccular dysfunction, with significantly higher rates of abnormal cervical VEMP (cVEMP) than ocular VEMP (oVEMP) (138). This selectivity reflects the greater vulnerability of otolith organs—particularly the saccule, owing to its simpler vascular supply—to ischemic damage during microvasculopathy and endolymphatic hydrops. The frequent coexistence of abnormal cVEMP and low-frequency hearing loss further supports a common microcirculatory pathogenesis affecting both cochlear and vestibular end-organs.

Glucocorticoids remain the cornerstone of first-line therapy, with additional immunosuppression titrated to disease severity: mild cases are typically treated with corticosteroids alone or combined with intravenous immunoglobulin (IVIg), mycophenolate mofetil, or rituximab, whereas moderate-to-severe disease usually requires cyclophosphamide or tacrolimus (139). The benefit of adjunctive immunosuppressants remains uncertain; available evidence suggests that neither additional immunosuppressive agents nor IVIg significantly reduces relapse rates, arguing against their routine use in Susac syndrome (140).

### 3.9 Sarcoidosis

Sarcoidosis is a multisystemic inflammatory disorder of unknown etiology, pathologically characterized by non-caseating epithelioid cell granulomas within affected tissues (141, 142). Neurosarcoidosis (NS) occurs when granulomatous inflammation involves the central or peripheral nervous system, affecting an estimated 5% to 13% of patients with sarcoidosis (143). The disease exhibits significant heterogeneity, primarily affecting adults between 20 and 55 years of age. Incidence rates are notably higher, and the clinical course more intractable, among African American and Northern European populations (144). Diagnosing NS remains complex, requiring the integration of clinical manifestations with high-resolution neuroimaging and histopathological confirmation. Gadolinium-enhanced magnetic resonance imaging (MRI) is the preferred modality for identifying neurological involvement. It typically reveals leptomeningeal enhancement, cranial nerve hypertrophy, or intraparenchymal mass-like nodules (145). Concurrently, cerebrospinal fluid (CSF) analysis often demonstrates characteristic lymphocytic pleocytosis and elevated protein levels, reflecting an active neuroinflammatory state (146, 147).

Cochleovestibular nerve involvement (CVNI) is a rare yet debilitating manifestation of neurosarcoidosis (NS). Approximately 90% of affected patients present with vertigo, imbalance, or hearing loss as the primary clinical manifestation (148). Immunological analyses suggest that this dysfunction stems from the antigen-driven aberrant polarization of CD4+ helper T cells into Th1 and Th17.1 phenotypes. This process drives the substantial accumulation of pro-inflammatory cytokines, specifically TNF-α, IL-12, and IL-18, within the perineural microenvironment (149–151). Histopathological studies demonstrate extensive lymphocytic infiltration surrounding the eighth cranial nerve and its vasa nervorum. This characteristic, “perineural cuffing,” directly induces axonal degeneration and severe segmental demyelination (152). In addition to direct infiltrative injury, immune-mediated microvasculitis may occlude the inner ear microcirculation. Such occlusion facilitates secondary ischemic degeneration of vestibular end-organs, including the semicircular canal cristae (153). The clinically observed dissociation between vertical and horizontal semicircular canal function supports a coupled mechanism of selective immune-mediated injury to vestibular nerve branches and vasculogenic damage (154).

In patients with neurosarcoidosis (NS) manifesting as vestibular vertigo, the prompt initiation of immunosuppressive therapy is critical to prevent permanent functional disability. Glucocorticoids, such as prednisone or prednisolone, remain the first-line therapeutic standard. These agents bind to cytosolic receptors to inhibit multiple pro-inflammatory pathways, yielding significant improvements in auditory and vestibular function in approximately 70% of patients with cochleovestibular nerve involvement (CVNI) (155). For steroid-refractory or intractable cases, second-line agents—including methotrexate (MTX) and azathioprine (AZP)—control persistent inflammation by disrupting the metabolic cycles and clonal proliferation of aberrant lymphocytes. Targeted biological therapies represent a significant advancement in precision medicine for NS. Infliximab, a potent TNF-α antagonist, neutralizes both soluble and membrane-bound TNF-α to disrupt molecular signals essential for granuloma maintenance (156). Clinical evidence demonstrates its efficacy in inducing significant regression of severe neurological lesions and intractable hydrocephalus (157). Furthermore, Janus kinase (JAK) inhibitors (e.g., tofacitinib) and mTOR pathway regulators are currently being investigated for their potential to induce macrophage metabolic reprogramming and reverse the pathological mechanisms of refractory neuroinflammation (158, 159).

### 3.10 Rheumatoid arthritis

Rheumatoid arthritis (RA) is a chronic, systemic autoimmune inflammatory disease characterized by persistent synovial involvement. Its pathophysiological progression typically entails synovial hyperplasia, progressive cartilage degradation, and subchondral bone erosion (160–162). Globally, RA prevalence ranges from 0.24% to 1% (163). The disease exhibits marked gender heterogeneity, with women being two to three times more susceptible than men (164). Clinical diagnosis relies on the 2010 ACR/EULAR classification criteria. These criteria assess the count and anatomical distribution of affected joints, serological autoantibody titers (e.g., RF and ACPA), acute-phase reactant levels (CRP and ESR), and symptom duration. While joint-related disability is the primary disease burden, RA frequently manifests with complex extra-articular involvement affecting the cardiovascular, respiratory, and vestibular systems. Consequently, vertigo arising from vestibular dysfunction has emerged as a prominent atypical clinical presentation in RA patients (165).

The precise mechanism of audiovestibular dysfunction in rheumatoid arthritis (RA) involves a multidimensional immune cascade. This process is primarily driven by autoantibody-mediated attacks and cytokine-induced microenvironmental dysregulation. Specifically, Type II collagen serves as a core autoantigenic target during RA progression. It is a vital matrix component found in inner ear hair cells, the tectorial membrane, and the crista ampullaris. High-titer Type II collagen autoantibodies promote the formation of *in situ* immune complexes within inner ear tissues. These complexes trigger localized inflammatory necrosis, nuclear degeneration of hair cells, and mechanical collapse of the semicircular canals, establishing the immunopathological basis for vertigo (166). Furthermore, elevated pro-inflammatory cytokines (e.g., IL-6 and TNF-α) and matrix metalloproteinases (e.g., MMP-3) can cross the blood-labyrinth barrier (167, 168). Sustained IL-6 levels correlate with systemic bone erosion and promote oxidative stress-induced damage to vestibular receptors (166). Additionally, RA-associated microvasculitis may affect the inner ear’s nourishing vessels (*vasa vasorum*), leading to localized ischemic degeneration (169, 170).

Immunotherapy for rheumatoid arthritis (RA)-associated vestibular damage aims to preserve delicate inner ear architecture and facilitate functional compensation by disrupting inflammatory cascades. Conventional synthetic disease-modifying antirheumatic drugs (csDMARDs), primarily methotrexate (MTX), remain the therapeutic cornerstone by modulating immune cell metabolism (171). For patients refractory to csDMARDs, biological agents (bDMARDs)—specifically TNF-α inhibitors (e.g., etanercept) and IL-6 receptor antagonists (e.g., tocilizumab)—exhibit significant otoprotective efficacy by neutralizing mediators of hair cell apoptosis (172). Emerging small-molecule Janus kinase (JAK) inhibitors, including tofacitinib and baricitinib, intracellularly intercept signaling pathways to truncate the transduction of multiple pro-inflammatory cytokines (173). In patients with high autoantibody titers, the CD20-targeted agent rituximab depletes B cells, thereby precluding the deposition of anti-Type II collagen antibodies and subsequent *in situ* immune complex formation. Glucocorticoids serve as essential bridging therapies, leveraging genomic regulatory mechanisms to downregulate inflammatory gene expression and mitigate acute vestibular edema and vertigo (174, 175).

### 3.11 Necrotizing vasculitides with polyangiitis

Necrotizing vasculitides with polyangiitis (GPA, formerly Wegener’s granulomatosis) are a heterogeneous systemic necrotizing vasculitis. Pathologically, it involves small-to-medium vessels, necrotizing granulomatous inflammation, and microvascular-mediated glomerulonephritis (176, 177). Global prevalence estimates for GPA range from 20 to 160 cases per million, while the annual incidence fluctuates between 0.5 and 20 cases per million (178, 179). The disease shows distinct ethnic and geographic patterns, with a higher incidence in populations of European descent and high-latitude cold regions (180). GPA affects all age groups but peaks between 65 and 74 years, showing a slight male preponderance in adults (181). Diagnosis integrates a clinical, serological, and histopathological triad. Approximately 80%–90% of patients test positive for antineutrophil cytoplasmic antibodies (ANCA), typically targeting proteinase 3 (PR3) (182). Otorhinolaryngological (ENT) manifestations are often the primary clinical presentation, affecting 70%–100% of patients through refractory chronic rhinosinusitis, serous otitis media, and progressive hearing loss (183, 184).

Although the incidence of vestibular dysfunction in patients with granulomatosis with polyangiitis (GPA) ranges from 8% to 65%, the associated disabling vertigo is often a crucial hallmark of inner ear involvement (185). Quantitative histopathological studies reveal that GPA exerts a cell-specific destructive effect on the vestibular neuroepithelium. Specifically, the density of type I vestibular hair cells is significantly decreased compared to age-matched controls, whereas type II hair cells are relatively spared (186). This damage is primarily driven by a PR3-ANCA-mediated immunological cascade. By specifically binding to PR3 on primed neutrophils, PR3-ANCA induces aberrant activation of the alternative complement pathway, generating high levels of the chemokine C5a. Binding of C5a to its receptor (C5aR) establishes a potent pro-inflammatory amplification loop. This loop drives neutrophil degranulation and the release of reactive oxygen species (ROS) and lysosomal proteases, ultimately causing necrotizing vasculitis within the vestibular microcirculation (187, 188). Furthermore, temporal bone studies corroborate that inflammatory occlusion of the labyrinthine or anterior vestibular arteries can trigger ischemic necrosis or localized microhemorrhages within the vestibular system (189). These microhemorrhages manifest as distinct hemosiderin deposition in the ampullae, semicircular canals, and vestibular cavity.

Management of vestibular and systemic manifestations in granulomatosis with polyangiitis (GPA) now centers on precision immunomodulation. This strategy prioritizes inhibiting the inflammatory cascade via targeted biological agents to achieve sustained clinical remission (190). Induction therapy has shifted from conventional non-specific immunosuppression toward Rituximab, a monoclonal antibody directed against the B-cell surface antigen CD20. Mechanistically, Rituximab rapidly depletes pathogenic B-cell clones, arresting the production of proteinase 3 (PR3)-ANCA at its source. This action mitigates neutrophil-mediated vascular endothelial injury and the subsequent ischemic necrosis of vestibular hair cells (190). Furthermore, the approval of Avacopan, a selective complement C5a receptor antagonist, marks a significant clinical advancement in AAV therapy. This oral agent specifically inhibits C5a-induced neutrophil recruitment, chemotaxis, and activation. The ADVOCATE trial demonstrated that Avacopan is non-inferior to high-dose glucocorticoids for remission induction. Crucially, it significantly reduces steroid-related systemic toxicity, offering a viable strategy to minimize hormone dependence in managing vestibular vasculitis (183).

### 3.12 Giant cell arteritis

Giant cell arteritis (GCA) is a chronic, systemic granulomatous vasculitis primarily affecting individuals older than 50 years (191). Its hallmark pathology involves transmural inflammatory infiltration of large and medium-sized arteries. Peak incidence occurs between ages 70 and 80, with a marked female predominance (192). Pronounced geographic and ethnic variations suggest a complex interaction between environmental factors and genetic susceptibility, specifically the HLA-DRB1*04 allele (193). Clinical manifestations typically include new-onset headache, jaw claudication, and systemic inflammatory symptoms (194, 195). Furthermore, GCA is closely associated with polymyalgia rheumatica (PMR) (196). The diagnostic paradigm is shifting from the conventional gold standard of biopsy toward multimodal imaging assessment. Techniques such as ultrasound, which detects the “halo sign,” and 18F-FDG PET/CT facilitate early, precise phenotype identification by visualizing vessel wall inflammation and metabolic activity (197).

The pathogenesis of vestibular vertigo in giant cell arteritis (GCA) is driven by a spatially organized breakdown of arterial wall immune defenses, leading to microcirculatory ischemia. While vertigo is an atypical manifestation of GCA, it affects approximately 11.9% of patients (198). This symptom frequently indicates inflammatory occlusion of terminal vessels, including the vertebrobasilar system and the internal auditory artery (199, 200). Consequently, ischemia impairs both the vestibular nuclei and peripheral sensors. Objective vestibular dysfunction occurs in nearly 90% of GCA cases (201). Furthermore, 20.5% of patients develop comorbid benign paroxysmal positional vertigo (BPPV) due to ischemia-induced otoconia detachment. At the immunopathological level, inflammation initiates in the arterial adventitia (202). Here, CD64+ macrophages and Th17 cells stimulate a systemic inflammatory cascade by producing interleukin-6 (IL-6), IL-1β, and IL-23. These cytokines establish the biochemical basis for the signal fluctuations observed in early-stage vertigo. Within the media, granulocyte-macrophage colony-stimulating factor (GM-CSF) regulates multinucleated giant cells. These cells secrete matrix metalloproteinase-9 (MMP-9) and reactive oxygen species (ROS), resulting in internal elastic lamina fragmentation and smooth muscle necrosis. This process compromises the structural integrity required for effective vasoconstrictive regulation. In the arterial intima, CD163+ macrophages orchestrate a “response-to-injury” program by secreting vascular endothelial growth factor (VEGF) and platelet-derived growth factor (PDGF). These factors promote myofibroblast proliferation, leading to severe luminal occlusion. This mechanism underlies the “vascular-systemic dissociation” phenomenon, explaining why ischemia risk persists in patients with vestibular symptoms despite normal systemic inflammatory markers (203–206). Finally, impaired immune surveillance by regulatory T cells (Tregs) facilitates the local expansion of pathogenic T-cell clones. This immune failure exacerbates irreversible damage to the vestibular system (207).

Current immunotherapeutic strategies for vestibular involvement in giant cell arteritis (GCA) are transitioning from broad-spectrum glucocorticoid suppression toward precision modulation of specific molecular targets. Although glucocorticoids (GCs) remain the standard therapy for acute vestibular ischemia by inhibiting the adventitia-mediated IL-6/Th17 axis to manage systemic inflammation, they offer limited control over occlusive ischemia driven by intimal remodeling. Furthermore, despite an approximately 56% symptomatic recovery rate, long-term GC use is hindered by substantial systemic toxicity (208, 209). As a significant pharmacological advancement, the IL-6 receptor antagonist tocilizumab precisely targets IL-6 signaling. This intervention facilitates steroid-free clinical remission and restores the homeostatic function of regulatory T cells (Tregs), thereby suppressing the clonal expansion of pathogenic T-cell subsets (207). The novel selective JAK1 inhibitor upadacitinib mitigates the effects of multiple pro-inflammatory cytokines by blocking the JAK/STAT signaling pathway. This mechanism offers a robust therapeutic alternative for steroid-dependent or refractory patients presenting with vertigo (209). Concurrently, monoclonal antibodies targeting granulocyte-macrophage colony-stimulating factor (GM-CSF), such as mavrilimumab, inhibit medial macrophage activation. This approach aims to intercept critical pathological stages that lead to vascular structural degradation and functional impairment (210). Collectively, this stratified targeting strategy marks the transition of GCA-related vestibular injury management from empirical therapy to a new paradigm of precision immune intervention.

## 4 Conclusion

Immune-mediated cochleovestibular disorders constitute a heterogeneous disease spectrum spanning otology and rheumatology, encompassing isolated inner-ear syndromes (delayed endolymphatic hydrops, autoimmune Ménière’s disease, and selected cases of bilateral vestibulopathy) as well as cochleovestibular manifestations of systemic autoimmune diseases (systemic lupus erythematosus, multiple sclerosis, Hashimoto thyroiditis, Behçet’s disease, Vogt–Koyanagi–Harada disease, psoriasis, Cogan syndrome, Susac syndrome, Sarcoidosis, Rheumatoid arthritis, Necrotizing vasculitides with polyangiitis and Giant cell arteritis). Integrating contemporary clinical, histopathological, imaging, and genetic evidence, this review concludes that, despite a rapidly growing literature, the field remains constrained by three fundamental limitations: diagnostic circularity, low-grade evidence, and overdiagnosis. Beyond biallelic RFC1 expansions in CANVAS, no highly specific biomarker exists (28); high-dose systemic glucocorticoids remain the near-universal first-line therapy yet rely almost exclusively on uncontrolled retrospective case series, with randomised trials being scarce and methodologically inadequate (50),Confirmed AIED accounts for merely 6.2% of SSNHL cases, indicating that AIED is likely overdiagnosed within the vestibulocochlear-immune axis (211). Consequently, many patients are subjected to prolonged, high-toxicity immunosuppression with only marginal benefit.

Future progress requires the simultaneous pursuit of the following priorities: establishment of international multicentre prospective registries with mandatory biobanking and standardised core outcome sets; unbiased proteomic, metabolomic, and single-cell transcriptomic profiling of perilymph, endolymphatic sac, and temporal bone specimens to delineate true pathogenic pathways; adequately powered, placebo-controlled trials of early intensive immunosuppression restricted to biomarker-enriched cohorts; and rigorous separation of primary autoimmune inner-ear disease from cochleovestibular involvement secondary to systemic rheumatological conditions.

Only by achieving these shifts can the field move beyond its current paradigm of “steroid response equals diagnosis, case reports equal evidence” and advance toward an era of precise diagnosis and rational, evidence-based therapy.
